# Identification of taste cells and reduced taste‐related proteins in saliva correlate with the impaired taste sensitivity in long‐coronavirus disease

**DOI:** 10.1002/ctm2.70165

**Published:** 2025-01-02

**Authors:** Parul Patel, Shveta Jaishankar, Mythily Srinivasan

**Affiliations:** ^1^ Oral Health Research Institute Indiana University School of Dentistry Indianapolis Indiana USA; ^2^ Department of Oral Pathology, Medicine and Radiology Indiana University School of Dentistry Indianapolis Indiana USA

1

Dear Editor,

Although transient chemosensory impairments in viral infections are common, the unique features of the recent severe acute respiratory coronavirus‐2 (CoV2) pandemic are subjective reports of taste dysfunction (TD) and TD without olfactory disturbance.^1^ Further, TD lasting for months to years has been observed with varied prevalence amongst post‐CoV2 symptoms. Persistent dysgeusia even in adolescents who were asymptomatic during primary infection is an emerging concern that could impact the overall health.^2^, ^3^

Taste perception is mediated by continuously renewing specialized taste receptor cells (TRC) that are supported by non‐gustatory epithelial cells. An optimal ratio of taste cell renewal and loss is tightly regulated to maintain taste function.^4^ Chronic viral infections, even in the absence of continued presence of the virus could hamper progenitor cell renewal. A longitudinal study of CoV2‐infected individuals with prolonged TD showed positive staining for spike protein and reduced number taste cells in tongue papillae as late as seventeen weeks post‐infection.^5^ Further, concomitant dysbiosis and inflammation could accelerate the loss of taste cells contributing to TD.^4^

Saliva acts as a conduit of substances to taste cells that mediate specific taste perception. Consistently, proteins that facilitate the transport of tastants are reduced in the saliva of individuals with TD. Some salivary proteins also contribute to the cellular turnover and homeostasis. Gustin, an enzymatic protein may directly influence taste perception as a trophic factor, or by modulating the buffering environment around taste receptors.^6^ Sonic hedgehog (SHH) is a morphogenic protein, signalling through which promotes proliferation and differentiation of taste progenitor cells. Disruption of SHH pathways in viral infections could impair TRC proliferation and differentiation, and thereby interfere with taste perception.^7^ In this pilot study, we correlated the objective measures of taste sensitivity with the salivary proteins involved in taste perception and investigated the presence of taste cells in saliva to explore the biological indicators of TD in long‐coronavirus disease (long‐COVID) individuals.

We invited respondents from our survey amongst individuals with subjective complaints of TD following the CoV2+ test and with records of the initial date of positive testing. Figure 1A shows the demographic features of our study cohort of individuals with a history of CoV2+ test once (long‐COVID) or more than once (long‐COVID reinfection). All individuals completed the Waterless Empirical Taste Test (WETT) which consists of 53 paper strips, with four strips of increasing concentrations each of sucrose, citric acid, sodium chloride, caffeine, and monosodium glutamate or no stimulus.^8^ Six individuals with no CoV2+ test also completed the objective taste test. The low number in the no CoV2+ control group was due to difficulty in recruiting CoV2 unexposed individuals with test results, particularly since CoV2 infection was widespread and predominantly asymptomatic. We observed that the sweet and bitter tastes were most impaired and that 10% of individuals exhibited low taste scores for longer than two years in both groups (Figure 1A–C).

**FIGURE 1:**
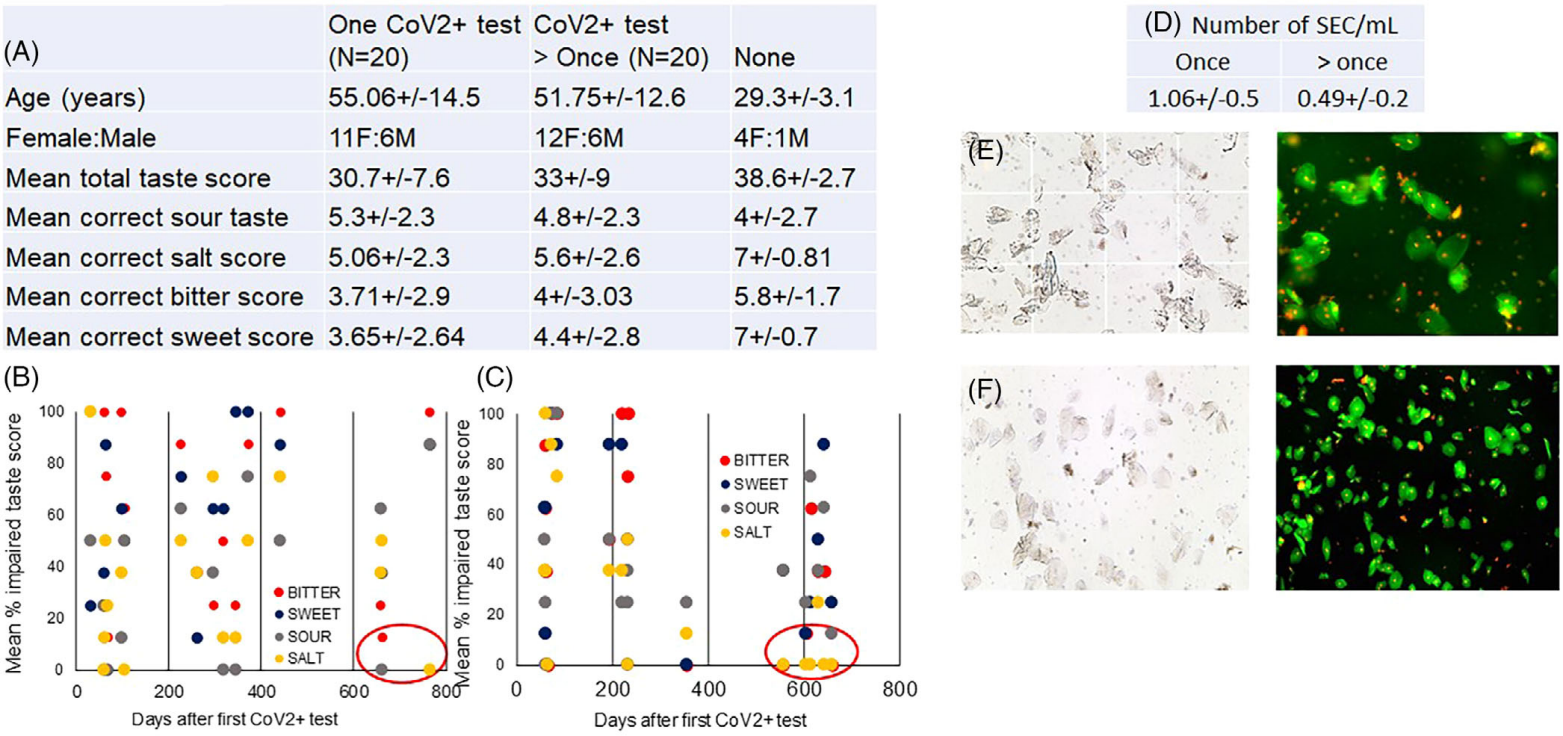
Taste dysfunction (TD) in long‐coronavirus disease (long‐COVID) individuals with or without CoV2 reinfection. (A) Table showing participant characteristics, mean total taste score and mean correct score of each taste type. Distribution of TD across the duration of CoV2+ test in (A) long‐COVID and (B) long‐COVID reinfection cohorts. (D) The table shows the number of epithelial cells (SEC)/mL of saliva. (E, F) shows representative SEC by brightfield and by acridine orange/propidium iodide staining in the two cohorts. The majority of SEC are viable as indicated by AO+ staining.

Since disruption of TRC homeostasis towards increased loss by exfoliation secondary to infection can lead to TD, we counted the epithelial cells in saliva (SEC). Previously the number of SEC has been shown to vary between 0.1 and 0.9 × 10^6^ cells/mL^9^. The average number of SEC was lower in long‐COVID reinfection than that in the long‐COVID cohort, although the difference was not statistically significant (Figure 1D). Acridine orange staining showed that > 70% of SEC were live cells (Figure 1E, F).

We evaluated the expressions of taste‐related proteins in clarified saliva by enzyme‐linked immunosorbent assay and in SEC by immunofluorescence. Salivary gustin and SHH exhibited an inverse relationship with the taste score in clarified saliva (Figure 2F, G). The SEC included cells staining positive for the epithelial marker pan‐cytokeratin (A), pan taste markers KCNQ1 (potassium voltage‐gated channel, subfamily Q, member 1) (Figure 2C) PLCβ1 (phospholipase C beta 1), (Figure 2D) and SHH (Figure 2E) as well as the neuronal marker SNAP25 (synaptosomal‐associated protein 25) (Figure 2B).

**FIGURE 2:**
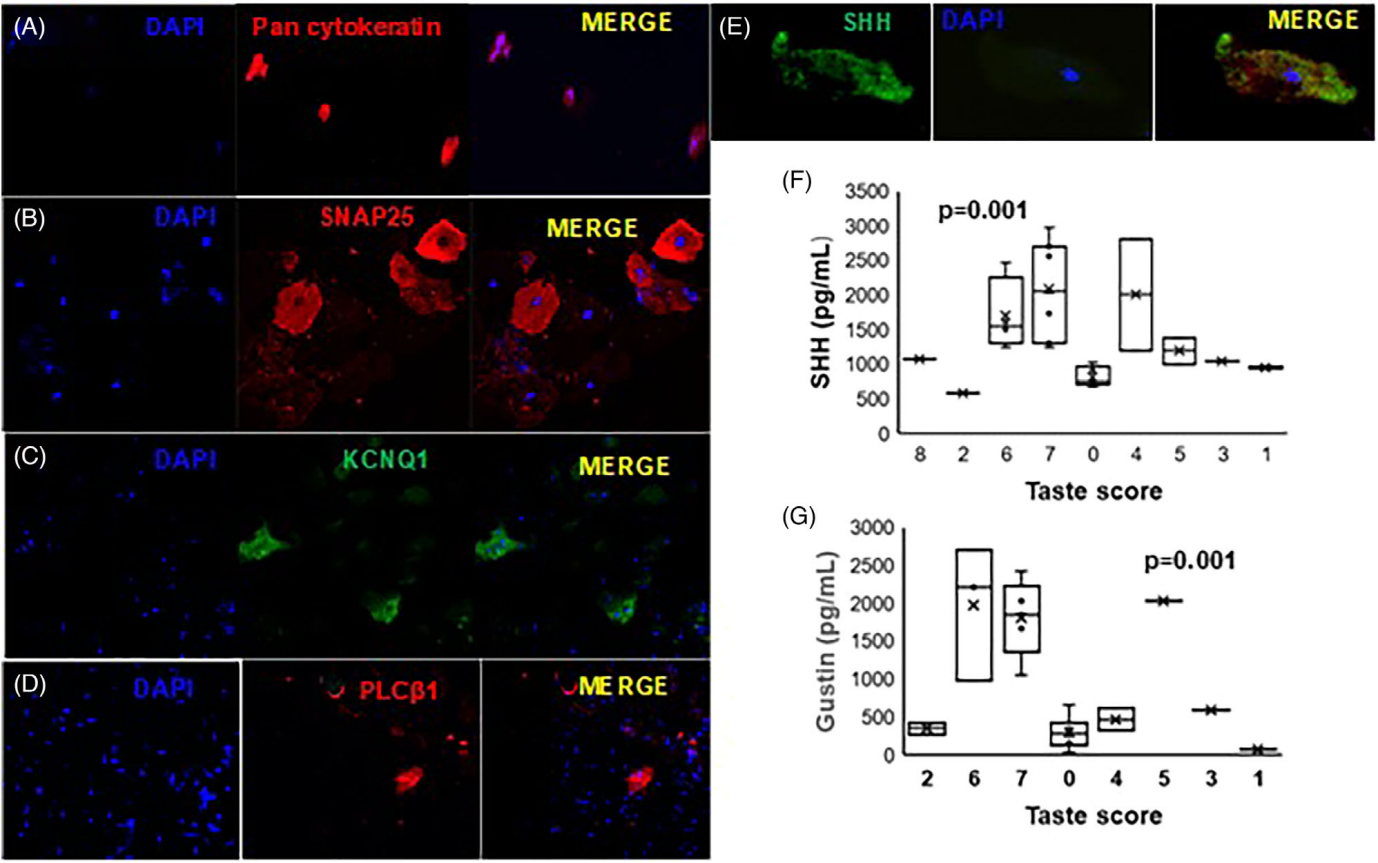
Clarified and cellular saliva express taste‐related proteins. Representative images of cells in saliva stained with (A) Pan cytokeratin, (B) SNAP25, (C) KCNQ, (D) PLCβ1, and (E) Sonic hedgehog (SHH). Nuclei are stained with Hoechst. Distribution of salivary SHH (F) and (G) gustin across the objective bitter score of the long‐coronavirus disease (long‐COVID) cohort. *p* < .001 as determined by the Wilcoxon rank test.

Since bitter and sweet were the most erroneously identified tastes, we determined the allelic expressions of two genes commonly associated with bitter taste perception. The *TAS2R38* gene encodes for receptors that mediate the perception of the bitter taste of PTC and 6‐n‐propylthiouracil. It exhibits three single‐nucleotide polymorphisms; the proline‐alanine‐valine (PAV/PAV) homozygotes experience intense bitter taste constituting “super‐tasters”; the alanine‐valine‐isoleucine (AVI/AVI) homozygotes are “non‐tasters” who do not taste bitter and PAV/AVI heterozygotes referred to as “tasters” experience intermediate bitter taste^10^. Similarly, rs2274333 (A/G) polymorphisms in the gustin gene are associated with bitter taste sensitivity^6^. Genetic analysis of 11 individuals in our cohort showed that with respect to *TAS2R38*, three were super‐tasters, two were non‐tasters and six were tasters. Gustin gene analysis showed that five were supertasters and six were tasters (Figure 3).

**FIGURE 3:**
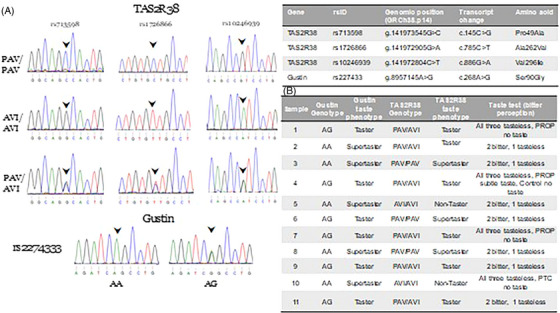
(A) Genotype distribution and (B) haplotype frequencies of polymorphisms of TAS2R38 and gustin gene and responses to the phenylthiocarbamide (PTC) test.

In conclusion, we report for the first time that cells expressing taste cell markers are observed in saliva and that taste‐related proteins are reduced in long‐COVID saliva correlating with lower bitter taste perception. Further, two of the three *TAS2R38* supertasters in our long‐COVID reinfection cohort exhibited the lowest bitter taste score, suggesting that the dysgeusia is more likely due to localized changes rather than genetic polymorphisms. The limitations of our study are the small sample size and lack of consideration of other long‐COVID symptoms. Nevertheless, our findings of variations in taste cells and related proteins in saliva open new avenues for investigating taste perception and its impact on health.

## AUTHOR CONTRIBUTIONS

P. P. Clinical study coordinator responsible for patient recruitment, chemosensory tests. S. J. Sample processing, experimental studies and data analysis. M. S. Hypothesis development, study design, data interpretation and manuscript preparation.

## FUNDING INFORMATION

This research was funded with support from “Delta Dental Research Program”.

## ETHICS STATEMENT

Institutional Review Board Statement: The study was conducted in accordance with the Decla‐ration of Helsinki and approved by the Institutional Review Board (or Ethics Committee) of Indiana University Purdue University at Indianapolis (protocol code 15239, approved May 25th, 2022) for the electronic survey study. Informed Consent Statement: Informed consent was obtained from all subjects involved in the study as part of the survey in accordance with the IRB approval.
